# Immunohistochemical Pattern of CD34 Distribution in Different Types of Basal Cell Carcinoma and in Peritumoral Skin

**DOI:** 10.3390/medicina62010158

**Published:** 2026-01-13

**Authors:** Vladimir Petrovic, Aleksandar Petrovic, Ivan R. Nikolic, Nataša Vidovic, Tijana Dencic, Ilija Golubovic, Miroslav Milic, Aleksandra Antovic

**Affiliations:** 1 Department of Histology and Embryology, Faculty of Medicine, University of Niš, Boulevard of Dr Zoran Đinđić 81, 18000 Niš, Serbia; aleksandar.petrovic@medfak.ni.ac.rs (A.P.); inikolic@junis.ni.ac.rs (I.R.N.); 2 Center for Pathology and Pathological Anatomy, University Clinical Centre Niš, Boulevard of Dr Zoran Đinđić 48, 18000 Niš, Serbia; natasa.vidovic71@gmail.com (N.V.); tijana.dencic29@gmail.com (T.D.); 3 Clinic for Digestive Surgery, University Clinical Centre Niš, Boulevard of Dr Zoran Đinđić 48, 18000 Niš, Serbia; 4 Department for Forensic Medicine, Faculty of Medicine, University of Niš, Boulevard of Dr Zoran Đinđić 81, 18000 Niš, Serbia; miroslav.milic@medfak.ni.ac.rs (M.M.); aleksantovic@yahoo.com (A.A.)

**Keywords:** basal cell carcinoma, CD34, peritumoral skin, juxtatumoral zone, transitional zone, marginal skin

## Abstract

*Background and Objectives*: Basal cell carcinoma (BCC) is the most common skin carcinoma, mainly occurring in older individuals. The aim of this study was to document the immunohistochemical distribution of CD34 in different histopathological types of BCC, as well as in the peritumoral and uninvolved skin of biopsy samples. *Materials and Methods*: Excisional biopsies of skin BCCs were routinely processed into paraffin blocks, and microtome sections were stained immunohistochemically for CD34. *Results*: A consistent finding in skin samples containing BCC was the absence of CD34 in the following extravascular structures: neoplastic cells, epidermis and its derivatives (except for the cells of the isthmic part of the outer hair follicle sheath), fibroblast-like cells of BCC tumor stroma, as well as in the papillary dermis in the tumor region. Fibroblast-like cells of the tumor stroma were variably CD34 immunopositive only in the nodular type of BCC. In all examined biopsies, part of the dermis adjacent to the BCC tumor mass (juxtatumoral zone) was characterized by pronounced CD34 immunopositivity. In the transitional zone of peritumoral skin and in marginal skin, CD34-positive connective tissue cells were observed in the periadnexal dermis around: sebaceous gland lobules, the secretory coils of eccrine sweat glands, the pilosebaceous canal, as well as in the perimysium of the arrector pili muscle. Fibrocytes of fibrous sheaths encasing the isthmic part of hair follicles were CD34 negative, interposed between highly positive epithelial cells of the outer hair follicle sheath and the fibroblasts of the local reticular dermis. The transitional zone and uninvolved skin contained CD34-positive fibroblast-like cells situated between secondary bundles of reticular dermis, as well as CD34-positive cell processes within these bundles. *Conclusions*: The observed pattern of CD34 positivity within the examined regions shows a specific distribution, providing insight into the adaptive responses of the skin to the tumoral process.

## 1. Introduction

Basal cell carcinoma of the skin (BCC) belongs to the group of non-melanoma skin cancers and represents the most common malignancy in Caucasians, with an epidemic incidence worldwide, affecting mainly the older individuals [1,2,3]. These carcinomas originate from the basal layer of interfollicular epidermis and occur most frequently on the skin of the face and neck, as well as on skin exposed to long-term solar irradiation [4,5,6].

Although BCCs exhibit a low malignant potential and very rarely metastasize, they display a wide range of organizational versatility of their tumor masses composed of neoplastic nests and tumor stroma, which makes them an interesting nosological category suitable for studies of epithelial–stromal relationships [7,8]. Furthermore, it is of great importance to gain insight into the effects that the tumor mass exerts on its immediate surroundings, i.e., the peritumoral skin. For these reasons, basal cell carcinoma of the skin, with its different histopathological types, is a good model for the investigation of rich morphological neoplastic epithelial–stromal interactions, as well as transformation and adaptation of neighboring skin structures.

The antigen CD34 (cluster of differentiation 34) is a transmembrane protein of the cell membrane that belongs to the family of single pass sialylated glycoproteins [9,10]. Since its discovery, it has been widely considered as a marker of stem and progenitor cells of hematopoiesis [9,11,12]. CD34 is also regarded as one of the “traditionally” most reliable indicators of endothelial cells of blood vessels, while its expression is absent from the endothelial cells of hepatic sinusoidal capillaries and lymphatic vessels [10,13]. Recently, CD34 has gained more significance in the analysis of the organization of fibroblast-like cell populations, in normal and pathologically altered tissues. The pattern of CD34 immunohistochemical distribution has been studied in various types of basal cell carcinoma as well, mainly as a differential diagnostic marker in relation to other epithelial skin tumors, and in the context of the histological architecture of the BCC tumor stroma [14,15,16,17]. The expression of CD34 has been evidenced recently in cells of both dermatological neoplastic structures and uninvolved skin [18,19,20].

Considering that the distribution of CD34 antigen may have significance in understanding the organizational and growth pattern of BCC tumor masses, and in elucidation of the adaptive mechanisms of uninvolved skin to tumor tissue, the aim of this research was to analyze the immunohistochemical presence and morphological distribution of CD34 antigen in: (1) tumor masses (tumor nests and tumor stroma) of different types of BCC, (2) peritumoral skin (juxtatumoral and transitional zone), and (3) structures of marginal, morphologically normal skin in the biopsies.

## 2. Materials and Methods

Material. The research material consisted of excisional biopsy samples of skin and subcutaneous tissue with basal cell carcinomas obtained from patients treated at the Clinical Centre—Niš. Each of the examined histopathological cases of BCC (*n* = 100) excised with the margin “into healthy tissue of skin” was taken from an individual patient, with the size of the examined tumors that ranged from 5 to 30 mm. All excised BCCs were primary, non-recurrent lesions, and none of the patients had metastases. The average age of patients included in the study was 69.5 ± 13.25 years (ranging from 34 to 87), with approximately equal gender distribution within each histopathological group. The analyzed BCCs were excised from the following anatomical locations: nasal region (*n* = 40), ear region (*n* = 8), upper lip (*n* = 2), forehead region (*n* = 7), temporal region (*n* = 20), cheek (*n* = 8), zygomatic region (*n* = 9), and skin of the back (*n* = 6). The exclusion criteria for the study were cases of BCC from the periocular region of the face, acral lesions, BCCs with a multicentric pattern of growth, recurrent lesions, and the presence of metastases. Therefore, for the purposes of this study, tissue samples were classified exclusively according to their histopathological type, having the 20 BCC cases in each of the following groups: (1) superficial type, (2) solid (circumscribed) type, (3) nodular type, (4) infiltrative type, and (5) basosquamous type.

Methods. For histopathological diagnostics, the excisional biopsy skin samples containing BCCs were fixed in 10% formalin, routinely processed into paraffin blocks and archived at the Centre for Pathology and Pathological Anatomy (Faculty of Medicine—Niš, Clinical Centre—Niš). From paraffin blocks, tissue sections were cut on microtome, then subjected to hematoxylin-eosin staining and immunohistochemical marking of CD34 for descriptive light-microscopic morphological analysis.

Immunohistochemistry. The deparaffinized tissue sections were brought to distilled water and incubated with trypsin solution for 90 min to achieve antigen unmasking, and then quenched with the aqueous 3% hydrogen peroxide solution for 10 min. After rinsing in phosphate buffer, the tissue sections were incubated overnight at 4 °C with a mouse monoclonal antibody against CD34 (Dako, M716501, dilution 1:50). The secondary antibody conjugated with horseradish peroxidase (Real EnVision System, Dako, K5007) was next applied for 30 min, and diaminobenzidine (DAB) was used as the chromogen, after which the slides were dehydrated, cleared in xylene, and mounted with Canada balsam and cover slips.

Descriptive analysis. Microscopic slides were analyzed using the Olympus BX50 light microscope (Olympus, Japan, Tokyo) equipped with a digital camera, Leica DFC295 (Leica Microsystems, Germany, Wetzlar), at the Department of Histology and Embryology, Faculty of Medicine, University of Niš.

**Figure 1:**
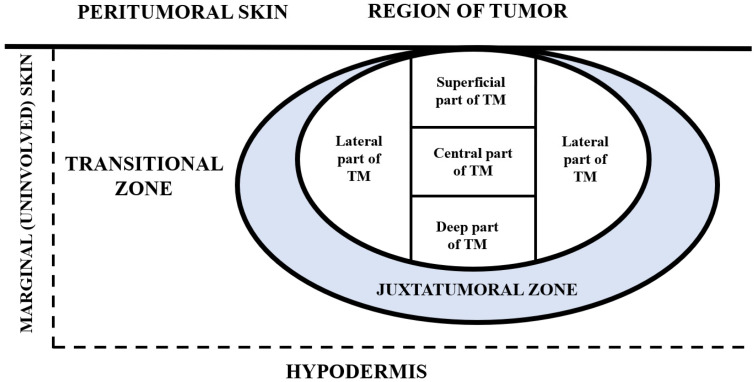
The examined parts of the BCC histological samples. TM stands for tumor mass, consisting of tumor nests and tumor stroma. The peritumoral skin was divided into two compartments juxtatumoral zone (directly surrounding the tumor mass) and transitional zone (the region of skin between the juxtatumoral zone and marginal skin). The morphologically normal and unalterated region of skin peripherally to the transition zone was analyzed as a marginal skin.

The analysis was performed in four regions of the examined tumors: superficial, central, deep, and lateral, as well as in the region of peritumoral skin and marginal (uninvolved) skin. Peritumoral skin was further divided into two zones: (1) juxtatumoral zone (part of skin biopsy that is in closest physical relation to BCC tumor mass) and (2) transitional zone (part of skin biopsy between the juxtatumoral zone up to the uninvolved skin at the periphery of skin biopsy specimen) (Figure 1).

Each of the examined cases had an “internal control” (except superficial BCCs), consisting of apparently healthy, morphologically unaltered marginal skin of the excision sample. For descriptive histological analysis, the following histological elements from the marginal skin area were included: adventitial dermis comprising papillary and adnexal dermis, reticular dermis, covering epidermis, hair follicle (infundibulum, isthmic region, hair bulb), pilosebaceous canal, sebaceous glands, secretory coils and excretory ducts of merocrine sweat glands, organization of vascular components, organization of neural components, as well as paracutaneous structures (hypodermis).

Seminquantitative analysis. The semiquantitative analysis was used to assess the intensity of CD34 immunopositivity in the juxtatumoral and transitional zones, as well as in the marginal skin. The scoring system was based on the intensity of the CD34-immunohistochemical reaction: absence of positivity—0, weak positivity—1, medium positivity—2, and strong positivity—3. Two independent researchers examined BCC samples, and their estimates were reviewed and reported as mean values. Standard deviations were also calculated. The numerical data were analyzed in SigmaStat 3.5 using the Kruskal–Wallis test. One slide from each sample was analyzed, so in total there were 100 examined slides (20 for each histopathological type).

## 3. Results

Besides intense CD34 positivity in endothelial cells of blood vessels, a consistent finding in tumor regions of all examined histopathological types of BCC was: CD34 negativity of BCC’s neoplastic cells, of overlaying regional part of epidermis, and of extravascular structures of the regional papillary dermis. Differences in extravascular CD34 expression were not observed between the compared regions of the BCC tumor mass.

**Figure 2:**
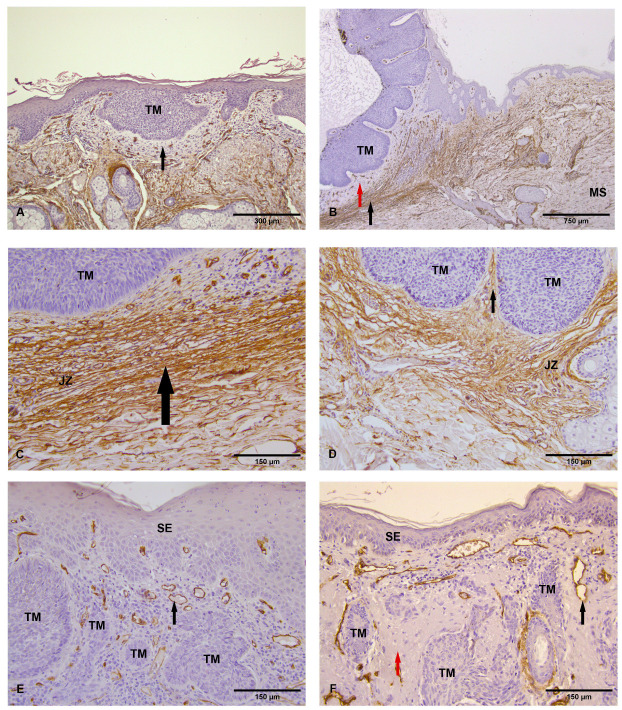
Expression of CD34 in different types of BCC. (**A**) Superficial BCC. Neoplastic cells in tumor mass (TM) and extravascular part of tumor stroma show the absence of CD34 immunopositivity (black arrow), ×100, (**B**) Solid-cystic BCC. Neoplastic cells of the tumor mass (TM) and extravascular part of tumor stroma (red arrow) surrounding them are devoid of CD34 immunopositivity. Juxtatumoral zone (black arrow) is present in the form of strongly CD34-positive belt encircling the tumor mass. Juxtamedular zone stands in strong contrast with the weaker positivity of the marginal skin (MS), ×40, (**C**) Compressed and atrophied collagen fibers (black arrow) of juxtatumoral zone (JZ) around the tumour mass in solid-cystic BCC, ×200, (**D**) Nodular BCC. Neoplastic cells of the tumor nests (TM) are CD34 immunogative. Tumor stroma (black arrow) and juxtatumoral zone (JZ) show strong immunoreaction to CD34, ×200, (**E**) Infiltrative BCC. Epithelial cells of surface epithelium (SE), neoplastic cells of tumor nests (TM), and extravascular part of the tumor stroma are CD34 immunonegative. The endothelial cells of the blood vessels in tumor stroma (black arrow) show strong CD34 immunoreaction, ×200, (**F**) Basosquamous BCC. Epithelial cells of surface epithelium (SE), neoplastic cells of tumor nests (TM), and extravascular part of the tumor stroma (red arrow) are CD34 immunonegative. The endothelial cells of the blood vessels in tumor stroma (black arrow) show strong CD34 immunoreaction ×200.

Superficial BCC. The induced perinodular tumor stroma did not exhibit immunoreactivity to the CD34 antigen. A frequent accompaniment of superficial BCCs was a moderately expressed CD34-negative lymphocytic–plasmacytic infiltrate of the surrounding tumor stroma. In histologically unaltered dermis, present beneath and around the tumor lesion, the staining pattern for the CD34 antigen corresponded to that of normal skin, like CD34 positivity of reticular dermis fibroblasts (Figure 2A).

Solid/cystic BCC. In solid/cystic BCCs, the tumor-induced stroma was relatively sparse compared to other examined histopathological types of BCC. Around the tumor mass, a thin band of unstained perinodular tumor stroma was observed, showing a clear contrast to the surrounding juxtatumoral zone, which was observed in the form of a compressed reticular dermis composed of 10–15 layers of CD34-immunoreactive flattened fibroblasts, arranged in a relatively parallel pattern, interposed between thin atrophic bundles of collagen fibers (Figure 2B,C). Immunoreactivity of dermis decreased gradually from this zone, being continued with the weaker CD34 immunoreactivity of the transitional zone of the reticular dermis, and towards marginal skin at the periphery of the samples (Figure 2B). Small blood vessels with CD34-positive endothelium were observed in greater numbers in the juxtatumoral and transitional zones compared to the neighboring histologically normal dermis of marginal skin.

Nodular BCC. The internodular and perinodular tumor stroma exhibited varying levels of CD34 antigen expression, as well as morphological diversity and differences in physical distribution across regions of the tumor mass. The tumor stroma was CD34 immunopositive in six out of twenty examined cases in this group (6/20), while in the rest of examined samples the extravascular CD34 immunopositivity was absent. In juxtatumoral zone, the reticular dermis surrounding the tumor mass demonstrated CD34 immunoreactivity. This reticular dermis, whose inner margin was in contact with the internodular induced tumor stroma of nodular BCCs, did not display the structural organization and clear demarcation observed in solid/cystic BCCs (Figure 2D).

Infiltrative BCC. The infiltrative type of BCC exhibited a markedly more prominent tumor stroma, whose perinodular and internodular compartments showed a monomorphic pattern of absence of extravascular CD34 immunoreactivity in the examined cases. The juxtatumoral zone demonstrated a lower degree of immunoreactivity to the CD34 antigen compared to solid/cystic and nodular BCCs, as well as a reduction in its structural organization. In certain cases, CD34 immunoreactivity was absent in the reticular dermis of the juxtatumoral zone, where only lymphoplasmacytic infiltrates or areas of CD34-negative refractory stroma were observed (Figure 2E).

Basosquamous BCC. In all samples of human basosquamous carcinomas, within the tumor mass and between the tumor nests, there was abundant tumor stroma of a reactive–proplastic type with relatively numerous large fibroblast-like cells that were CD34 negative. In these stromal areas, individual, relatively distant blood vessels were observed, whose endothelial cells were intensely positive for the CD34 antigen. The surrounding skin and subcutaneous structures showed contrast in their positivity against the irregular boundary formed by the CD34-negative stroma. Similarly to the infiltrative BCC, the juxtatumoral zone showed weaker CD34 immunopositivity and reduced structural organization. Typically, variations in depth and width within this description were practically absent, and the entire tumor mass presented in a monomorphic manner (Figure 2F).

**Figure 3:**
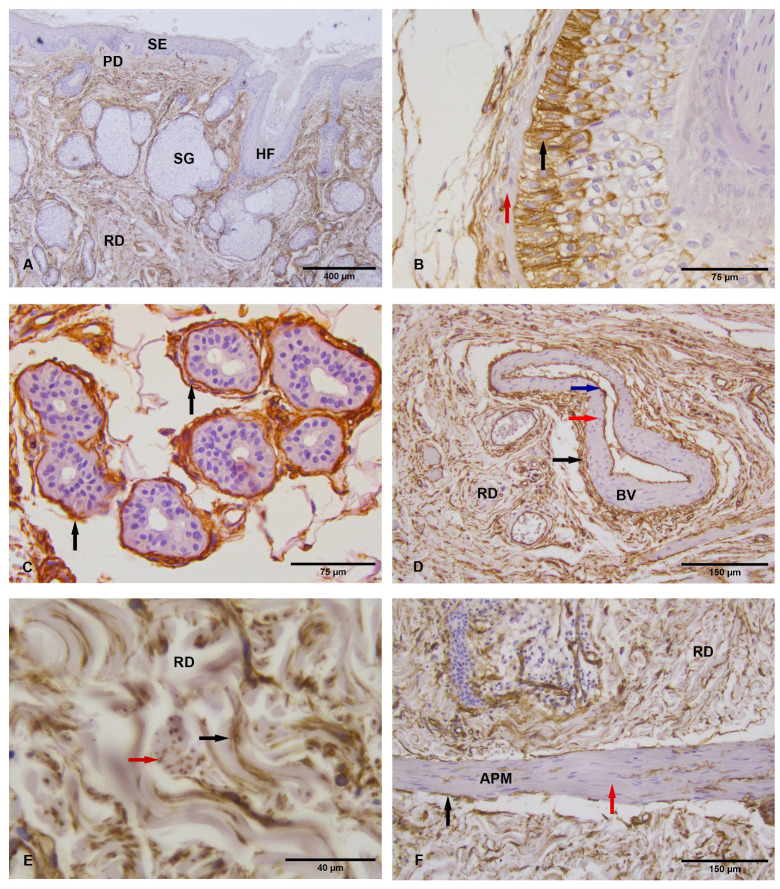
Expression of CD34 in marginal skin. (**A**) Panoramic view of CD34 immunoreactivity in marginal skin. SE—surface epithelium, PD—papillary dermis, HF—hair follicle, SG—sebaceous gland, RD—reticular dermis, ×40, (**B**) CD34 immunoreactive cells in outer root sheath of hair follicle (black arrow), and flattened CD34-immunonegative fibroblasts of the fibrous hair follicle sheath (red arrow), ×400, (**C**) CD34-immunopositive stromal cells (black arrow) around the coils of the merocrine sweat glands. The epithelial cells of merocrine glands do not show the immunopositivity on CD34, ×400, (**D**) CD34 immunopositivity in stromal cells of reticular dermis (RD) and in adventitia (black arrow) and endothelial cells of blood vessels (red arrow). The smooth muscle cells of blood vessels (blue arrow) are CD34 immunonegative. BV—blood vessel, ×200 (**E**) CD34 immunopositivity in fibrillar (black arrow) and oval structures (red arrow) in the interstitium of secondary bundles of collagen fibers in reticular dermis (RD), ×800, (**F**) CD34-immunopositive cells around the arrector pili muscle (black arrow) in the reticular dermis (RD). The smooth muscle cells do not express CD34 (red arrow). APM—arrector pili muscle, ×200.

CD34 immunoreactivity in transitional zone and marginal skin. The cells of the superficial epidermis of the transitional zone and the marginal (uninvolved) skin were immunohistochemically negative for CD34, as well as the epithelial cells of sebaceous and merocrine glands (Figure 3A). In larger hair follicles (scalp hair follicles), the only strong CD34 labeling was present in the cells of the isthmic part of the outer root sheath, while other cells were CD34 non-reactive (Figure 3B). In the papillary dermis, the extravascular structures were CD34 negative (Figure 3A). Strong extravascular CD34 immunopositivity in flattened fibroblasts of the periadnexal dermis was observed around coils of merocrine sweat glands, around the sebaceous gland’s lobules, and adjoined proximal parts of sebaceous excretory ducts (Figure 3A,C). The periadnexal dermis surrounding the infundibular, isthmic, and suprabulbar parts of hair follicles, together with the connective tissue of the hair root bulb papilla, showed the absence of CD34 positivity.

Between densely distributed secondary bundles of reticular dermis of marginal skin, numerous CD34-positive, spindle-shaped fibroblasts were observed. Interestingly, in the reticular dermis of transitional zone and marginal skin was the presence of CD34-positive cellular processes inside secondary bundles of reticular dermis (interposed between primary bundles of collagen fibers) with relatively uniform thickness, which depending on the plane of section, displayed oval or polygonal morphology (Figure 3E). Although mostly observed as independent structures at the level of cross-sections, their physical continuity with the CD34-positive spindle-shaped cells could be evidenced on tangential sections. The distribution pattern of this immunopositivity was observed in the whole reticular dermis (extending up to papillary dermis, periadnexal dermal sheaths, and until the boundary of adipose tissue of the hypodermis) and was partially preserved within the juxtatumoral zone.

Around the arrector pili muscle, a single layer of flattened fibrocytoid cells in the form of a perimysial sheath was observed, which were intensely immunoreactive to CD34 (Figure 2F).

CD34 immunoreactivity in hypodermis. CD34 immunopositivity was observed in stromal cells around unilocular adipocytes, in connective tissue septa surrounding clusters of adipose cells, in endothelial cells of small and large blood vessels, and in stromal cells of the adventitia of larger blood vessels (Figure 3D). In the neural structures in the hypodermis, CD34 positivity was seen in the endothelium of blood vessels within nerve fascicles, as well as in individual flattened fibroblasts of the perineurium. Additionally, around cross-sections of peripheral nerves, CD34 immunoreactivity was present in elongated and flattened fibroblasts of the surrounding stroma. Schwann cells and axons did not show CD34 labeling.

Semiquantitaive analysis. The results of the semiquantitative analysis of CD34 immunostaining intensity in the juxtatumoral zone show the highest values in solid/cystic BCC, followed by nodular and superficial BCC. In contrast, infiltrative forms of BCC (infiltrative and basosquamous BCC) display significantly weaker CD34 staining intensity. The statistically significant difference was found between superficial and basosquamous BCC, while the intensity of CD34 immunoreactivity in solid/cystic and nodular BCCs was significantly higher compared to infiltrative and basosquamous BCCs (Table 1).

The analysis of the transitional zone showed no statistical difference between the examined BCC types, whereas in the marginal skin, statistical significance was found between solid/cystic and infiltrative BCC (Table 1).

The statistical analysis of juxtatumoral, transitional and marginal skin within the examined histopathological types of BCCs revealed that the intensity of CD34 reaction in the juxtatumoral zone shows significantly higher values compared to marginal skin in superficial, solid/cystic, nodular and basosquamous BCCs (Table 1). In turn, the statistically significantly stronger CD34 reaction in the juxtatumoral compared to the transitional zone was observed in all examined types of BCCs (Table 1).

## 4. Discussion

General finding in all examined types of BCCs was the absence of CD34 positivity in neoplastic cells, and in extravascular structures of papillary dermis and tumor stroma. Also, a prominent or less structured demarcation of strongly positive CD34 juxtatumoral fibroblast-like cells was consistently present in the reticular dermis, with a gradual decline in CD34 positivity throughout the morphologically unaltered dermis toward the edges of the skin samples.

Without the exception, the neoplastic cells of all analyzed histopathological types of BCC were expectedly CD34 negative, which is concordant with the fact that well differentiated dermatologic neoplasms retain the potential to differentiate in boundaries of cytomorphology of originating cell type, displaying the same absence of CD34 expression as the cells of interfollicular epidermis in all regions of analyzed skin samples [14,16,17,21]. In support of this, the epithelial cells of epidermis and its derivatives are always CD34 negative, except for cells in the isthmic part of the hair follicle outer sheath, which show strong CD34 immunopositivity, also reported by other authors [22]. It should be emphasized that desmoplastic trichilemmomas, in contrast to BCCs, are composed of neoplastic cells that are strongly CD34 positive, retaining the expression of the originating, CD34-positive cell population of the hair follicle`s outer sheath [23,24].

In principle, the extravascular part of tumor stroma in all analyzed BCC types was not labeled with CD34 [14,16,17,23,25]. Exceptions were observed in parts of a few cases of nodular BCCs, in which extravascular CD34 immunoreactivity was detected in fibroblasts of the tumor stroma. This pattern is not unexpected given the close morphologic similarities between this type of BCC and trichoepithelioma. Kirchmann highlighted the differential diagnostic significance of the CD34 expression pattern in BCC versus trichoepithelioma, reporting that, compared with BCCs, neoplastic nests of trichoepithelioma are surrounded by CD34-immunopositive stroma [14]. This has been fully or partially confirmed in subsequent studies [16,17,23,25]. However, there are a number of articles reporting that BCCs’ tumor stroma, to a lesser degree, also may express extravascular presence of CD34, questioning the role of CD34 as a reliable marker for differential diagnosis between these two conditions [21,26]. Furthermore, in all examined types of BCCs (apart from nodular BCC), the morphological pattern of tumor stroma differentiation and its absence of CD34 extravascular positivity may be comparable with the pattern of CD34 expression and morphology of papillary dermis underlying neighboring epidermis, so the tumor stroma of BCCs may be seen as an “exaggerated” variant of papillary dermis.

Contrary to BCCs, in a number of more biologically aggressive carcinomas—including breast, lung, colon, prostate, pancreas, and thyroid cancers—as well as in some benign lesions such as benign prostatic hyperplasia, “recruitment” of CD34-immunoreactive fibroblasts into tumor stroma occurs [27,28,29,30,31,32,33]. Some authors have proposed a potential role for CD34 antigen expression as an indicator of tumor aggressiveness, noting decreased or absent CD34 positivity in the stroma of invasive ductal breast carcinomas compared with in situ ductal carcinoma or normal skin [34,35,36]. Certainly, the etiopathogenetic mechanisms underlying the development and progression of a particular neoplastic lesion are complex and multifactorial. The characteristics of tumor stoma reflect the patterns of its originating microanatomic complexes, and as cancer cells further dedifferentiate, stromal differentiation also declines. The essential role of vascular factor for stromal development, which defined the concept of “reactive vasculature”, was seen as a necessary condition for the transformation of normal into tumor stroma. Increased microvascular density in BCCs has been shown to correlate with the aggressive behavior of these neoplasms, suggesting it may serve as a potential mechanism for growth and metastasis [37,38]. However, in BCCs, the originating pattern of tumor stroma differentiation may be the closest to that of papillary dermis.

The mechanism underlying the absence of CD34 positivity in stromal cells of invasive tumor stroma remains unknown. It may be hypothesized that the activity of certain enzymes, such as matrix metalloproteinases, may contribute to the removal of CD34 molecules from tumor stroma [39,40]. Furthermore, under the influence of tumor growth factor β1, one of the main “activators” of normal fibroblasts, CD34 expression is lost and replaced by the expression of alpha-smooth muscle actin (α-SMA) in fibroblasts [35,36,41,42]. Newly formed α-SMA-positive myofibroblasts are presumed not to retain the immunological functions of CD34-positive fibroblasts from which they originated, nor their capacity for stromal remodeling and regulation of angiogenesis, which may significantly influence further tumor progression and dissemination [34,36,43,44]. Although this proposed mechanism could explain the absence of CD34-positive fibroblasts in the stroma of more aggressive lesions, it remains hypothetical and requires confirmation through further experimental studies.

In available literature, the skin surrounding the tumor masses is usually referred to as the peritumoral skin. However, based on our observation, the peritumoral skin should be analyzed through two separate compartments: the juxtatumoral zone and transitional zone, in order to provide a more precise analysis of histopathological samples of BCCs. Juxtatumoral skin represents the first or imminent response of skin structures to the BCC and is distinguishable as a separate region compared to the rest of skin structures positioned peripherally in the rest of the biopsy skin samples. It represents the biological phenomenon of sharp transition between the tumor masses and the adjacent dermis. Compared to the pattern of CD34 expression in the region of tumor, the juxtatumoral zone of reticular dermis shows strong CD34 immunohistochemical positivity in stromal cells and microvascular blood vessels in all examined BCCs. Juxtatumoral zone may be seen as a reaction of the reticular dermis against the neoplastic tissue of BCC. Situated around the BCC tumor mass, it appears in the form of a compressed reticular dermis composed of 10–15 layers of CD34-immunoreactive flattened fibroblasts, arranged in a relatively parallel pattern and interposed between thin flattened bundles of collagen fibers, and numerous blood vessels. Occasionally accompanied by a chronic inflammatory infiltrate containing lymphocytes and plasma cells, this zone may be understood as a reaction of the skin, particularly the reticular dermis, to the presence of a developing neoplastic mass. Juxtatumoral zone is always present and displays the strongest and densest CD34 immunopositivity compared to the rest of the skin samples. This is primarily caused by the endothelial CD34 positivity in a multitude of small blood vessels, as well as by numerous CD34-positive fibroblast-like cells present in this zone. The impression of CD34 density is further alleviated by the reduction in interposed reticular dermis collagen bundles. Juxtatumoral CD34 positivity shortly decreases toward the transition zone of reticular dermis, where the seemingly normal dermal morphology exists, and whose CD34-positive fibroblasts appear as more dispersed between relatively wide secondary bundles of collagen fibers. However, the intensity of CD34 staining gradually decreases towards the uninvolved, marginal skin at the periphery of the samples. Juxtatumoral zone, according to its composition, closely resembles structurally to reparatory granulation tissue. Our results suggest that the juxtatumoral zone shows a more pronounced structural organization and very strong CD34 immunopositivity in superficial and solid/cystic BCC, where this zone completely encircles the tumor mass and separates it from adjacent reticular dermis. However, in infiltrative and basosquamous BCCs, the juxtatumoral zone shows a reduced structural organization and weaker CD34 immunopositivity. Juxtatumoral zone in nodular BCC, although strongly CD34 immunopositive showed less structural organization compared to superficial and solid/cystic BCCs.

The extravascular CD34 immunopositivity was absent in papillary dermis in the tumor region, as well as in the peritumoral and marginal skin in all examined types of BCC. Some authors suggest that the CD34-immunopositive stromal cells found in the adventitia of blood vessels, around the hair follicle, sebaceous, and merocrine glands, as well as around the arrector pili muscle, are not fibroblasts but belong to the category of telocytes [45,46]. However, these claims should be taken with precaution and need to be further addressed by using appropriate immunohistochemical markers and electron microscopy.

The presence of CD34-positive elongated structures interposed within the secondary bundles of collagen fibers in the reticular dermis of marginal skin may be considered as protoplasmatic structures, corresponding to cellular processes of interfascicular fibroblasts [20]. Given that the plasmalemmas of adjacent connective tissue cells in interfascicular spaces are also CD34 positive, we assume that these elongated, protoplasmic structures are cellular parts of these cells [22]. This finding may indicate the existence of one extensive cellular network of fibroblast-like cells whose cellular bodies are situated between secondary bundles of fibroelastic connective tissue, and whose elongated cellular processes extend through the structure of secondary bundles, interconnect, and form the continuous cytoreticulum in the reticular dermis. Such a CD34-positive cellular network in the reticular dermis may represent a specific and separate region of cutaneous immune surveillance or defense by inflammatory response and may also be involved in extravascular migratory activities of immunocompetent cells or in modulating their activities.

Additionally, CD34 immunoreactivity in the reticular dermis was also observed in flattened perifollicular cells situated parallel to the isthmic part of hair follicles, whose outer sheath cells also show strong CD34 positivity [22,47,48]. These CD34-positive flattened cells are separated from the outer sheath cells by interposed CD34-negative, exceptionally elongated fibrocytes of the fibrous sheath of the hair follicle. The CD34-negative fibrocytes of the fibrous hair follicle sheath are more likely to play the role of telocytes compared to neighboring CD34-positive fibroblasts of the reticular dermis [45]; however, further studies are needed to confirm this hypothesis.

Since CD34 mediates cell adhesion, it may be particularly important in stabilizing the stromal CD34-positive reticular network and thereby counteracting tumor cell migration and tissue invasion [9]. Also, the available data suggest that the expression of ICAM-1 and VCAM-1 in fibroblasts, which are generally absent or low, can be upregulated by inflammatory cytokines, raising the question of their possible role in tumorigenesis and tumor spread in skin cancers [49,50,51]. The origin of CD34-positive fibroblasts in reticular dermis and partially in periadnexal dermis remains a subject of investigation, and further studies are needed to elucidate their role in skin cancers completely.

## 5. Conclusions

The results of the study show a distinct pattern of CD34 positivity across the examined regions of the skin samples. The absence of CD34 in extravascular structures of the tumor mass stands in clear contrast with the pronounced CD34 positivity seen in the stromal cells of juxtatumoral and transitional zone of the peritumoral skin, as well as moderate in the marginal, unaltered skin. The juxtatumoral zone can be seen as an adaptive response of the adjacent skin, manifesting as a compressed reticular dermis containing highly CD34-positive stromal cells that surround the tumor masses. Reticular dermis of the marginal skin showed the presence of numerous CD34-positive fibroblast-like cells around the secondary bundles of collagen fibers, and whose CD34-positive cellular processes may be seen inside the bundles. Bearing in mind the roles of CD34, the observed CD34-positive cytoreticulum in the reticular dermis should be further studied in the light of its immunological and protective functions of adjacent skin against neoplastic processes.

## Figures and Tables

**Table 1:** Semiquantitative score of distribution of CD34 immunoreaction intensity in analyzed zones of skin in biopsies of different histopathological types of BCC.

	Juxtatumoral Zone	Transitional Zone	Marginal Skin
Superficial BCC	2.2 ± 0.9 ^a,g,q^	1.9 ± 0.4 ^q^	1.7 ± 0.4 ^g^
Solid BCC	2.9 ± 0.2 ^b,c,h,i^	1.7 ± 0.3 ^h^	1.5 ± 0.4 ^f,i^
Nodular BCC	2.5 ± 0.5 ^d,e,j,k^	1.9 ± 0.6 ^j^	1.7 ± 0.3 ^k^
Infiltrative BCC	1.4 ± 0.9 ^b,d,l^	2.0 ± 0.4 ^l^	1.9 ± 0.5 ^f^
Basosquamous BCC	1.1 ± 0.8 ^a,c,e,m,n^	2.0 ± 0.5 ^m^	1.8 ± 0.4 ^n^

Numerical data are presented as average values ± standard deviation. Letters in superscript mark the statistically significant difference for *p* < 0.05. For statistical analysis Kruskal–Wallis test was used.

## Data Availability

The data presented in this study are available on request from the corresponding author.

## Abbreviations

The following abbreviations are used in this manuscript:

BCC	Basal cell carcinoma
CD34	Cluster of differentiation 34
α-SMA	Alpha smooth muscle actin
ICAM-1	Intercellular adhesion molecule 1
VCAM-1	Vascular cell adhesion molecule 1
